# Oral Health Disparities in Dementia and Marginalized Communities: Determinants and Interventions

**DOI:** 10.1093/geroni/igaf122.1022

**Published:** 2025-12-31

**Authors:** Xiang Qi, Weiyu Mao, Bei Wu

## Abstract

This symposium presents a comprehensive examination of oral health disparities in older adults, with a focus on those with dementia and from marginalized communities. The first presentation employs machine learning (ie., XGBoost) to analyze social and physical environmental determinants of edentulism across 38,379 census tracts, identifying social vulnerability and climate zones as key predictors. The second study investigates oral health-related quality of life among Chinese American dementia caregivers, revealing that financial hardship and fewer natural teeth are associated with poorer outcomes. The third presentation evaluates a care partner-assisted intervention using behavioral change techniques to improve oral hygiene in individuals with mild dementia, demonstrating the potential of structured coaching. Finally, in the same randomized controlled trial, the authors assessed the effectiveness of a smart toothbrush intervention with and without personalized coaching, showing sustained improvements in plaque and gingival indices with coaching. Together, these studies highlight the need for tailored, community-based approaches to address oral health disparities and showcase innovative methodologies for gerontological research. By integrating advanced analytics, mixed-methods, and clinical trials, this symposium offers robust evidence to inform interventions and policies aimed at improving oral health equity for vulnerable older adults. Oral Health Interest Group Sponsored Symposium

